# Pollen defenses negatively impact foraging and fitness in a generalist bee (*Bombus impatiens*: Apidae)

**DOI:** 10.1038/s41598-020-58274-2

**Published:** 2020-02-20

**Authors:** Kristen K. Brochu, Maria T. van Dyke, Nelson J. Milano, Jessica D. Petersen, Scott H. McArt, Brian A. Nault, André Kessler, Bryan N. Danforth

**Affiliations:** 1 Present Address: Department of Entomology, Penn State University, University Park, PA USA; 2 Department of Entomology, Cornell University, Ithaca, NY USA; 3 Minnesota Department of Natural Resources, St. Paul, MN USA; 4 Department of Entomology, Cornell AgriTech, Cornell University, Geneva, NY USA; 5 Department of Ecology and Evolutionary Biology, Cornell University, Ithaca, NY USA

**Keywords:** Animal behaviour, Animal physiology, Entomology, Behavioural ecology

## Abstract

Plants may benefit from limiting the community of generalist floral visitors if the species that remain are more effective pollinators and less effective pollenivores. Plants can reduce access to pollen through altered floral cues or morphological structures, but can also reduce consumption through direct pollen defenses. We observed that *Eucera (Peponapis) pruinosa*, a specialist bee on *Cucurbita* plants, collected pure loads of pollen while generalist honey bees and bumble bees collected negligible amounts of cucurbit pollen, even though all groups of bees visited these flowers. Cucurbit flowers have no morphological adaptations to limit pollen collection by bees, thus we assessed their potential for physical, nutritional, and chemical pollen traits that might act as defenses to limit pollen loss to generalist pollinators. Bumble bee (*Bombus impatiens*) microcolonies experienced reduced pollen consumption, mortality, and reproduction as well as increased stress responses when exposed to nutritional and mechanical pollen defenses. These bees also experienced physiological effects of these defenses in the form of hindgut expansion and gut melanization. Chemical defenses alone increased the area of gut melanization in larger bees and induced possible compensatory feeding. Together, these results suggest that generalist bumble bees avoid collecting cucurbit pollen due to the physiological costs of physical and chemical pollen defenses.

## Introduction

Many studies attribute the vast diversity of bees to their mutualistic pollenivorous lifestyle, yet bee-plant interactions are much more complex than mutualisms. The conflict in bee-plant interactions arises because bees (Apoidea) can consume vast quantities of pollen, but vary markedly in effectiveness as pollinators^1,2^. Thus, bees are more accurately described as highly specialized and extremely efficient herbivores, while also acting as pollinators. Consequently, plants must balance their need to be pollinated against the loss of pollen from foraging bees.

If pollen loss is detrimental, plants may have evolved various ways to limit this loss. Many plants use volatile cues as exclusive channels of communication to attract specific pollinators, frequently specialists^3,4^. Specialist bees that visit a restricted set of plant genera or species are generally assumed to be the most efficient pollinators^5^, but they can also exact a heavy toll through their highly efficient pollen removal^6^. Plants have also evolved various morphological adaptations that limit pollen collection, such as hidden anthers^7,8^, or poricidal anthers that require specialized behaviour (i.e. buzz-pollination) to release pollen^1,9^. Finally, plants can also reduce pollen consumption directly through pollen traits that act as defenses, such as chemical compounds^10–13^, physical properties^14,15^, and even a lack of essential nutrients^16–20^. Strategies that reduce the size of the community of possible floral visitors can benefit plants provided that they increase the fidelity and effectiveness of the remaining pollinators^1,21^.

If plants are chemically or physically defending their pollen, we might expect specialist bee species to have physiological adaptations that allow them to feed exclusively on their preferred host pollen, but this same pollen source could be indigestible or nutritionally inadequate for a range of generalist species^22–25^. Physiological restrictions could be caused by nutritional requirements not served by single-plant diets, a lack of mechanisms to deal with pollen defenses that interfere with digestion, reproduction, or growth processes, or even direct toxic effects^10,26–30^. These strategies to limit pollen consumption are not mutually exclusive^31^, but little is known about the relative costs of investing in each strategy. Studies on potential pollen defenses generally consider these traits in isolation, confounding physical or chemical traits with effects of poor nutrition^19,27,28^, demonstrating deterrence without actual consumption costs^14,32,33^ or, more rarely, toxic effects that do not translate to changes in foraging behaviour^10^. Several studies have examined the effects of defended nectar on pollinators^34–36^; however, there is surprisingly little work examining how the nutritional quality of pollen and its physical or chemical properties intersect to impact bee health and fitness, especially considering how important pollen defenses could be in determining patterns of bee foraging and health.

Squash and pumpkin (genus *Cucurbita*) have unisexual flowers, requiring pollinators to set fruit. They have a diverse pollinator fauna of both host-plant generalists (*Bombus*, *Apis*, *Melissodes*, *Lasioglossum*, *Agapostemon*, and *Halictus*) and narrow host-plant specialists (*Eucera [Peponapis]* and *E. [Xenoglossa]*) across their geographic range^37–41^. Cucurbit flowers have no morphological adaptations to limit pollen collection, thus physical or chemical pollen traits could act as defenses for cucurbit plants to limit pollen loss to generalist, low-fidelity visitors. Cucurbit pollen is large, spiky, has a sticky pollenkitt and contains many chemical compounds, which could all act as defenses^42^. It also has a lower protein:lipid ratio than that generally preferred by *Bombus impatiens*, a common generalist visitor, which could render it a non-preferred diet for that species^19,20,30^. Cucurbit specialist bees, like *Eucera (Peponapis) pruinosa*, thrive on a solely cucurbit pollen diet, despite its potential pollen defenses; however, it is unknown if generalist pollinators, such as the common eastern bumble bee (*Bombus impatiens*), collect large quantities of cucurbit pollen, or are negatively impacted by feeding on a diet of cucurbit pollen. Accordingly, we evaluated the effects of pollen traits on the foraging behaviour and physiology of generalist pollinators, which largely overlap with the specialist pollinators in their geographical and seasonal range, in this system.

Our first objective evaluated the frequency of cucurbit pollen collected by generalists (honey bees, *Apis mellifera;* and bumble bees, *Bombus spp*.), often used for commercial pollination of cucurbits, in the field. We predicted that generalist pollinators would collect fewer cucurbit pollen grains compared with other plant families. Our second objective examined fitness costs for a generalist pollinator feeding on cucurbit pollen. We predicted that a generalist would suffer increased fitness costs by consuming cucurbit pollen. Cucurbit pollen may exhibit three levels of defenses: chemical traits (secondary plant metabolites), physical traits (large size or spines) and poor nutrition (a lack of essential nutrients). Our study was designed to distinguish among the effects of specific pollen traits as well as their combined impact. We predicted that a generalist would suffer reduced fitness when consuming a diet with pollen traits that acted as defenses. We predicted that bees would incur these costs as a combination of increased mortality, reduced or inefficient resource utilization, reduced reproduction, and increased stress responses.

## Results

### Pollen collection

**Figure 1:**
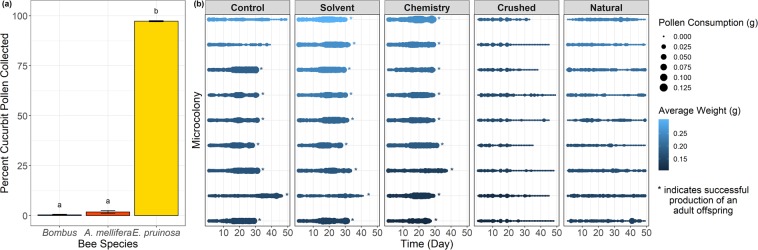
*Cucurbita* pollen use in field and lab experiments. (**a**) Percentage of *Cucurbita* pollen grains observed in typical pollen loads collected by three bee species located in cucurbit fields. These field studies were conducted in New York in 2011 and 2012 (*A. mellifera* and *B. impatiens*) and 2014 (*E. pruinosa*). Error bars represent standard errors. (**b**) Untransformed pollen consumption by *B. impatiens* in microcolonies over time. Each horizontal line represents the consumption values for a single microcolony over time. The thickness of the line indicates the average value of pollen consumption for each bee in the microcolony on that date. The colour of each line indicates the average weight of the microcolony (which does not change over time), arranged vertically (top to bottom) from lightest to heaviest microcolonies. Asterisks indicate when data collection for a microcolony was terminated due to the successful production of an adult offspring. No asterisk indicates lack of offspring survival to adulthood.

We found a significant effect of species on the proportion of cucurbit pollen collection (*Χ*^2^_(2)_ = 112.11, p < 0.001). Post-hoc tests show that *E. pruinosa* carried significantly more cucurbit pollen than both generalist species (*B. impatiens* and *A. mellifera*), which collected minimal cucurbit pollen (Fig. 1a). All *E. pruinosa* individuals actually carried pure cucurbit pollen loads, which is considered as consisting of 90% or more of one type of pollen^25^. *E. pruinosa* well exceeded the minimum threshold to be considered cucurbit specialists, with a minimum of 93% cucurbit grains, and an average of 97%, with the remainder made up of other Cucurbitaceae pollen. In contrast, cucurbit pollen made up a small percentage of the total pollen collected by the generalist *A. mellifera* and *B. impatiens*, only 2.0% and 0.4% respectively. Pollen types represented by less than 3% of the sample are generally considered to be ‘accidental contact’ and are not normally recorded as host-plant pollen^43,44^. Only 2.0% and 0.3% of *A. mellifera* and *B. impatiens* bees sampled, respectively, had cucurbit pollen in quantities greater than 3%, suggesting that few bees are actively collecting cucurbit pollen.

### Effect of pollen traits on microcolony performance

#### Resource utilization

We found a significant effect of day (F_(1,1671)_ = 86.304, p < 0.001), diet treatment by day (F_(4,1669)_ = 20.741, p < 0.001), day by average weight (F_(1,1670)_ = 81.280, p < 0.001), and diet treatment by day by average weight (F_(4,1669)_ = 18.658, p < 0.001) on pollen consumption by *B. impatiens* (Fig. 1b). Post-hoc analyses indicated that *B. impatiens* pollen consumption increased over time for all diet treatments except the Crushed and Natural treatments. Microcolonies fed the Control, Solvent, and Chemistry treatments increased their pollen consumption over time for all average weights, except for lighter microcolonies fed the Solvent treatment and heavier microcolonies fed the Control treatment. As the experiment progressed, heavier microcolonies fed the Solvent and Chemistry treatment increased their pollen consumption more than lighter microcolonies. The reverse trend was true for microcolonies fed the Control treatment, whereby the lighter microcolonies increased their consumption more over time than heavier microcolonies. This is likely due to two heavy microcolonies fed the Control treatment that never produced adult offspring, while small microcolonies increased pollen use to provide for their offspring. Microcolonies fed the Crushed and Natural treatments tended to decrease pollen consumption over time. Microcolonies with higher average weights consumed more sucrose per bee per day (F_(1,14)_ = 16.001, p = 0.001, Fig. S1).

#### Mortality

**Figure 2:**
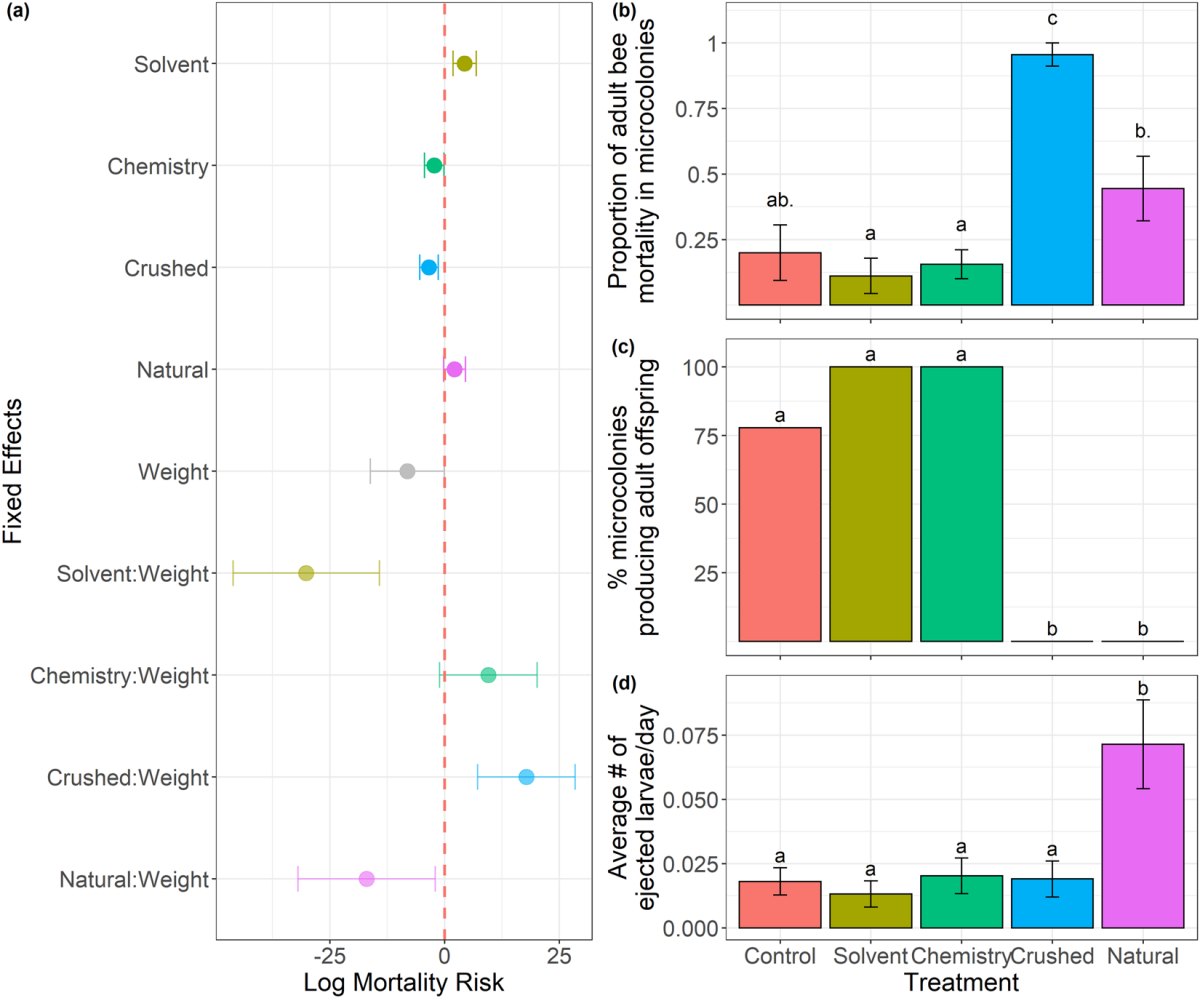
Fitness effects on *B. impatiens* in microcolonies. Letters indicate significance at p < 0.05, with a ‘ . ’ after the letter indicating a marginal difference at p < 0.1 for that comparison. (**a**) Log Mortality Risk based on Cox Proportional Hazards Mixed-Effects model coefficients with standard error bars. Error bars that do not cross the red dotted line indicate significant effects at p < 0.1. Interactive effects can be interpreted as increased or decreased risk for a given treatment with increasing weight, thus we observe a higher mortality risk with increasing weight in the Crushed treatment, and a lower mortality risk with increasing weight in the Solvent treatment. (**b**) Overall proportion of mortality in each microcolony across treatments. (**c**) Percentage of microcolonies producing adult offspring across treatments. (**d**) Average number of larvae ejected per day across treatments.

We found a significant effect of treatment (*Χ*^2^_(4)_ = 13.427, p < 0.01) and of treatment by weight (*Χ*^2^_(4)_ = 13.329, p < 0.01) on mortality over time. In post-hoc tests, compared to bees fed the Control treatment, there was a trend (p < 0.1) for increased mortality in bees fed the Solvent treatment and in larger bees fed the Crushed treatment. There was a trend (p < 0.1) for decreased mortality risk in bees fed the Crushed treatment, and in larger bees fed on both the Solvent and Natural treatments (Fig. 2a). We then assessed if the predicted mortality hazard was greater than zero after 27 days for each treatment across seven weight classes (minimum, 5^th^ percentile, 25^th^ percentile, median, 75^th^ percentile, 95^th^ percentile, maximum), with pairwise post-hoc tests and a Holm^45^ correction for multiple comparisons. Smaller bees (below the 25^th^ percentile) fed the Solvent and Natural treatment had a higher mortality risk (p < 0.05), and a trend (p < 0.1) for higher mortality risk at the 25^th^ percentile weight. Bees fed the Control treatment had a trend (p < 0.1) for higher mortality risk for weights at and below the median. Bees fed the Crushed treatment had a trend (p < 0.1) for higher mortality risk for weights at and above the 75^th^ percentile.

We found a significant effect of treatment (*Χ*^2^_(4)_ = 33.408, p < 0.001) on the proportion of worker mortality in microcolonies. A post-hoc Tukey test showed that microcolonies fed the Crushed treatment exhibited higher overall mortality than microcolonies fed all other treatments (p < 0.001), except the Natural treatments, while microcolonies fed the Natural treatment exhibited significantly and marginally higher overall mortality than microcolonies fed the Chemistry (p < 0.05) and Control (p < 0.1) treatments, respectively, but did not differ from microcolonies fed the Solvent diet (Fig. 2b).

#### Reproduction

While all microcolonies produced eggs, we found that treatment significantly affected the probability of a microcolony rearing their offspring to adulthood (Fisher’s Exact test, p < 0.001). Post-hoc Fisher exact pairwise comparisons with an FDR correction^46^ showed that microcolonies fed Control, Solvent, and Chemistry treatments were more likely to produce adult offspring than microcolonies fed Crushed and Natural cucurbit treatments, which never produced adults (Fig. 2c). Because microcolonies fed Crushed and Natural cucurbit treatments never produced adult offspring, we restricted all following analyses of reproduction to microcolonies that produced adult offspring. We found no significant effects of treatment or average weight on the number of days to the first eclosed offspring, the number of eclosed offspring per bee per day, or the average eclosed offspring weight.

#### Stress responses

When adult bees are stressed or their larvae are sick, they will eject larvae from their brood cells and discard them (where they defecate)^47,48^. Due to overdispersion in our data we fit a negative binomial distribution to assess the number of ejected larvae per day, with the log of the number of days of worker bee activity as an offset. We found a significant effect of treatment (*Χ*^2^_(4)_ = 16.804, p = 0.002, Fig. 2d), with post-hoc Tukey analyses showing that microcolonies fed the Natural cucurbit treatment ejected more larvae than microcolonies in all other treatments (p < 0.05). Another stress response evaluated was pollen diet efficiency, which is the number of eclosed offspring divided by the total pollen consumed per microcolony. Therefore, lower diet efficiency indicates that bees need to consume more pollen to produce each eclosed offspring, and could be due to an inability to adequately digest pollen or assimilate nutrients from their diet. We found a significant effect of treatment (F_(2,14)_ = 5.310, p = 0.019, Fig. S2) on pollen diet efficiency, with microcolonies fed the Chemistry treatment having a lower pollen efficiency than bees in the Control treatment (p < 0.005); however, they did not differ from the Solvent treatment, suggesting a possible subtle effect of the chemistry plus solvent combination that merits further investigation. This analysis was restricted to microcolonies that produced adult offspring.

**Figure 3:**
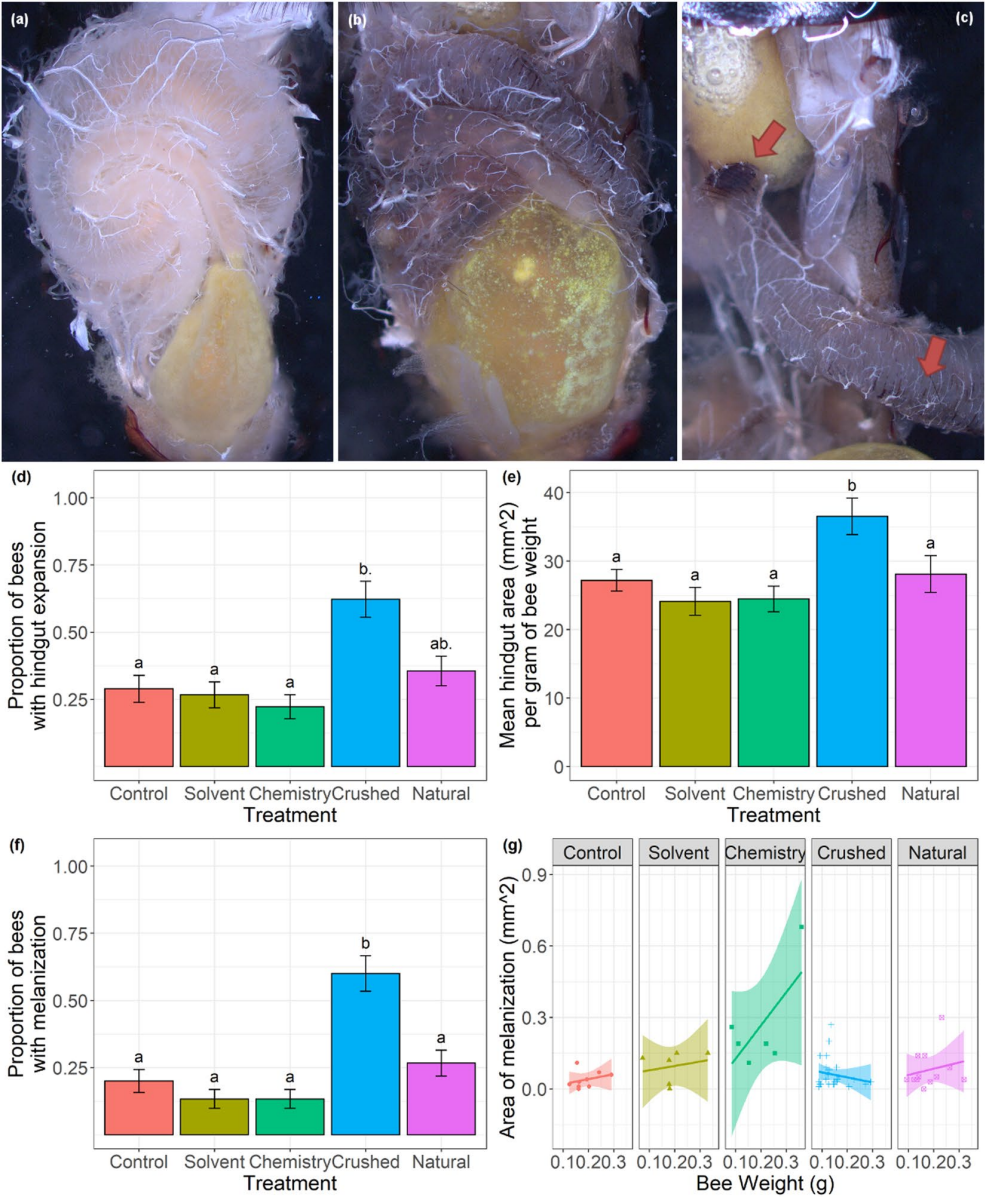
Signs of physiological stress on adult *B. impatiens* fed various diet treatments. Letters indicate significance at p < 0.05, with a ‘ . ’ after the letter indicating a marginal difference at p < 0.1 for that comparison. (**a**) Dorsal view of a normal bee gut from the Control treatment. (**b**) Dorsal view of a bee with hindgut expansion from the Chemistry treatment. The yellow hindgut can be observed to be swollen with respect to the coiled midgut. (**c**) Dorsal view of a bee with melanization (indicated by red arrows) on the midgut from the Chemistry treatment. (**d**) Overall proportion of bees exhibiting hindgut expansion across treatments. (**e**) Mean hindgut area (mm^2^) per gram of bee weight. (**f**) Overall proportion of bees exhibiting gut melanization across treatments. (**g**) Area of melanization (mm^2^) in each treatment across bee weights.

We defined physiological stress as hindgut expansion or gut melanization as observed in our dissections (see Fig. 3a–c for photographic examples). We found a significant effect of treatment on the proportion of bees exhibiting hindgut expansion (*Χ*^2^_(4)_ = 19.087, p < 0.001). Post-hoc tukey tests showed that bees in the Crushed treatment were significantly more likely to have an expanded hindgut than bees in all other treatments, except for bees in the Natural treatment, where they were only marginally (p < 0.1) more likely to exhibit this physiological stress (Fig. 3d). Quantitative analysis of hindgut expansion compared the area of the hindgut per gram of bee weight for all bees in the study. We found a significant effect of treatment (F_(4,210)_ = 5.432, p < 0.001) on the area of the hindgut per gram of bee weight. Post-hoc Tukey tests showed that bees fed the Crushed treatment were significantly more likely to have larger hindguts per gram of bee weight than all other treatments (Fig. 3e). Due to the large number of zeros in our dataset we analyzed the proportion of microcolonies exhibiting gut melanization separately, and then subsequently restricted the quantitative analysis to microcolonies that exhibited this sign of physiological stress. We found a significant effect of treatment (*Χ*^2^_(4)_ = 31.148, p < 0.001) on the proportion of bees exhibiting gut melanization. Post-hoc Tukey tests showed that bees fed the Crushed treatment were significantly more likely to exhibit gut melanization than all other treatments (Fig. 3f). We found a significant interaction of treatment and weight (F_(4,47)_ = 3.125, p = 0.023) on the area of melanization present. Post-hoc tukey tests compared treatment effects across seven weight classes (minimum, 5^th^ percentile, 25^th^ percentile, median, 75^th^ percentile, 95^th^ percentile, maximum). At and above the 25^th^ weight percentile, bees in the Chemistry treatment exhibited significantly more melanization than bees in the Control and Crushed Treatments. At and above the 50^th^ weight percentile, bees in the Chemistry treatment exhibited significantly more melanization than bees in all other treatments (Fig. 3g).

## Discussion

Our study explicitly assessed the interacting impacts of physical, chemical, and nutritional pollen traits on the foraging behaviour and fitness of a generalist pollen forager. We addressed this question via a pollen foraging study in the field and a mechanistic lab experiment. We observed that *Eucera (Peponapis) pruinosa*, a specialist bee on cucurbit plants, collected pure loads of cucurbit pollen in contrast to generalist honey bees and bumble bees which collected minimal amounts of cucurbit pollen, although all bee species visit cucurbit flowers (see^41^ for relative visitation rates). Bumble bee microcolonies fed non-cucurbit pollen increased pollen consumption over time, while microcolonies fed natural and crushed cucurbit pollen decreased consumption over time. Microcolonies fed crushed cucurbit pollen had higher mortality overall, with large bees in this treatment particularly at risk. Microcolonies fed natural and crushed cucurbit pollen never reared offspring to adulthood, while all other treatments always produced adult offspring, except for two microcolonies fed the control diet. Additionally, more larvae were ejected from microcolonies fed natural cucurbit pollen than in any other treatment. Finally, bees in the Crushed cucurbit pollen treatment were more likely to exhibit hindgut expansion and melanization, while larger bees fed the Chemistry treatment had more melanization than bees fed all other treatments. Together, these results suggest that *Bombus impatiens* workers avoid collecting cucurbit pollen due to the physiological costs associated with the consumption of pollen with multiple levels of defenses.

In the field, *B. impatiens* and *A. mellifera* foraged for pollen away from where their colonies were located in cucurbit fields. Only a very small percentage of cucurbit pollen grains were found within their corbiculae. Yet these generalist bees spent time foraging in cucurbit flowers, presumably for nectar^37,41,49^. This foraging behaviour is important because it suggests that bees recognize cucurbit flowers as a nectar source, but distinguish that the pollen should be avoided, likely using multi-sensory cues^15,50^. This collection avoidance means that as bees fail to groom pollen attached to their body into their corbiculae, more is available for transport and pollination, and in fact, bumble bees have been found to deposit the most pollen on a per visit basis in cucurbit systems, as compared to squash and honey bees^37^. This phenomenon could result in an increase in the amount of pollen successfully transferred and a corresponding increase in plant fitness^13^.

There are several mechanisms that could explain why generalist bees avoid collecting certain pollens^51–53^. Pre-ingestive effects (*e.g*. physical defenses, cues correlated with poor nutrition, chemical defenses, or chemical tastes) deter feeding (changing consumption behaviour), while post-ingestive effects, which can be pre-digestive (*e.g*. bees have different capacities for pollen digestion or toxins reduce nutrient digestibility) or post-digestive (*e.g*. a diet has insufficient nutrients or bees cannot metabolize diet toxins), can reduce growth and reproduction through malnutrition or direct toxic effects.

Pre-ingestive effects were evident in our study with reduced pollen consumption per bee in microcolonies fed both Crushed and Natural cucurbit pollen treatments, compared with microcolonies fed all other diet treatments, and a tendency to decrease pollen consumption over time. Reduced feeding in the cucurbit pollen treatments could be a response to a poor diet, or an attempt to minimize consumption of plant defenses in the diet^52,54,55^. Several studies have suggested that animals fed single-plant diets will do worse than on mixed diets, but this generalization does not apply to specialist bee species, which have evolved to feed exclusively on a small set of plant species. In contrast, generalist species may increase their feeding to compensate if a diet lacks nutrients, and reduce consumption if the diet contains toxins^54,55^; however, this pattern does not always hold if the poor diet is lacking in some essential nutrient or contains the wrong ratio of nutrients^20,52^. This pattern is further complicated by possible synergistic interactions between digestibility reducers and direct toxins, whereby compensatory feeding due to digestibility reducers would result in the increased consumption of a toxin, potentially leading to even lower ultimate consumption^56^. In another study, *B. impatiens* fed *C. pepo* pollen gained less weight over the seven day study period than bees fed other single-pollen diets or a multi-floral diet, and some bees in this treatment actually lost weight over the study period^19^. These results suggest that *C. pepo* pollen is nutritionally deficient for bumble bees, although this study showed no effect of *C. pepo* pollen on mortality or oocyte development, although this could be due to the shorter study time. Our pattern of reduced pollen consumption over time both supports the finding that cucurbit pollen is nutritionally deficient for *B. impatiens*, and suggests that pollen traits acting as defenses may deter feeding.

Our results on pollen consumption over time also provide some evidence for post-ingestive effects. Microcolonies of larger average weights fed the Solvent and Chemistry treatments consumed more pollen over time than smaller microcolonies in the same treatment (with microcolonies fed the Chemistry treatment consuming more pollen than microcolonies fed the Solvent treatment at all weights), but did not produce more or larger offspring, suggesting compensatory feeding to overcome a pre-digestive constraint whereby some component of the diet reduced digestibility^52,53,57^. We would expect this effect to be more pronounced in larger microcolonies which would need to consume proportionally more pollen than smaller microcolonies. Larger bees fed the Chemistry treatment also exhibited significantly more area of melanization in their gut, which would imply a post-digestive effect of chemical defenses on the gut.

While our results suggest no negative effects of chemical traits alone on microcolony performance, we believe they merit further investigation. Due to the termination of microcolonies once a new offspring was reared to adulthood, microcolonies fed the Chemistry treatment were not exposed to the treatment for as long as the Crushed and Natural treatments. If that had been the case, we may have observed a greater proportion of bees in the Chemistry treatment exhibiting gut melanization, mortality, or other fitness effects. In addition, our chemical extraction method can only succeed in isolating a subset of the potential chemicals found in cucurbit pollen, thus both cucurbit pollen treatments would have a more complex suite of chemicals which could have contributed to the stronger effects in these treatments. Our results suggested that while there may be subtle effects of chemical defenses on pollen efficiency, there are unlikely to be strong mortality effects due to toxins in pollen alone. Since most studies on toxins in pollen fail to account for the interacting effects of nutrition^12,27–29,31,58^, the strongest effects may occur when toxins interfere with digestion in an already poor nutritional diet, as in our cucurbit treatments^19,57,59,60^.

Post-ingestive effects in *B. impatiens* were also observed in our study in the form of reduced reproduction and increased mortality. Bees in the Crushed and Natural cucurbit treatments were unable to rear any offspring to adulthood, microcolonies fed Natural cucurbit pollen ejected more larvae, and microcolonies fed Crushed cucurbit pollen had higher proportions of mortality. If *B. impatiens* simply cannot digest cucurbit pollen this effect would be pre-digestive^52^, but since we observed severe negative effects in both Crushed and Natural cucurbit pollen treatments, we hypothesized that these effects are primarily post-digestive, where cucurbit pollen is either missing essential dietary components and/or has physical or chemical properties that interfere with physiological processes.

Interestingly, adults and larvae responded differently to our treatments, with adult bees in the Natural treatment not exhibiting greater mortality than controls, but with no larvae surviving to adulthood. This pattern suggests life-stage specific physiological adaptations. Other studies have also found that digestion is affected by the age of the animal, indicating that larval and adult digestion may differ^17,61^. Adult bees are capable of reducing their own diet consumption, but they may continue to provide larval bees with a set amount of food, which could have increased larval exposure to pollen defenses. This could explain the increased rates of larval ejection in our Natural cucurbit pollen treatment. Some of these larvae were blackened in appearance, suggesting that they were already sick when ejected, but some of the larvae appeared to be healthy, suggesting that their removal was more likely due to stress in the worker bees^47,48^. This observation supports the hypothesis that deleterious effects of the diet on the adult bees themselves could have contributed to reduced reproduction in our cucurbit treatments. Malnutrition or increased physiological costs of metabolizing toxins can reduce investment in producing or caring for offspring^48,57^. All of these effects may have worked in concert to prevent any larvae in the Crushed or Natural treatments from completing their development to adulthood.

We also found size-specific differences in mortality. Smaller bees fed the Solvent control and Natural cucurbit pollen had higher mortality risks, suggesting that they could have been suffering from increased toxic effects as a result of their small size^62,63^. Microcolonies fed the Natural cucurbit pollen treatment exhibited high levels of larval ejection and high mortality risk only for small bees. The amount of pollen consumed by larval bumble bees determines their size as adults; therefore, smaller adult bees may already have sub-optimal health^64,65^. Larger bees also tend to be more dominant, and sometimes restrict access to food for smaller bees^62,66^, which could cause smaller bees to become stressed and even more susceptible to the effects of pollen defenses^63^. Interestingly, this effect did not extend to bees in the Chemistry treatment. If this effect is mediated by a weakened immune system in small bees, it is possible that the Chemistry treatment bolstered the immune system, negating the mortality effects of the Solvent (DMSO) alone. Conversely, microcolonies fed the Crushed cucurbit pollen treatment exhibited high adult mortality, particularly for large bees. Larger bees have been shown to be less resilient to nectar shortages, likely due to a decreased proportion of lipid tissues compared to smaller bees^67,68^. This effect could explain our pattern of larger bees in the Crushed treatment having a higher risk of mortality, if our manipulation of the crushed cucurbit pollen had exacerbated effects due to malnutrition^67,68^. Especially in combination with physical or chemical defenses, the Crushed treatment may have proven difficult to overcome for the adult bees.

While our study was designed to provide evidence for the effects of both malnutrition and physical or chemical pollen traits, due to the unexpected strength of the responses in both Crushed and Natural cucurbit treatments, particularly the increased mortality in the Crushed cucurbit treatment, it is difficult to parse out the mechanisms of these effects. Such high mortality in the Crushed cucurbit treatment was unexpected as we predicted that the Natural cucurbit pollen would have the most severe effects on both adults and offspring. It is possible that in crushing the cucurbit pollen, we changed the pollen by: (1) increasing evaporation and drying of the pollen thus reducing its nutritional value, (2) increasing the level of chemical defenses by releasing additional chemicals contained within the exine or released as a result of a breakup of compartmentalization, that would otherwise have been inaccessible, or (3) increasing the level of physical resistance by creating smaller shards of exines that had a more severe effect than the intact exine. Our pollen consumption data doesn’t provide support for the nutritional hypothesis because we failed to find differences in pollen consumption between microcolonies fed the Crushed and the Natural treatments, but our mortality data for bees fed the Crushed treatment is consistent with malnutrition having a greater effect on larger bees. Our dissection data was also able to shed some light on these different hypotheses. We found that bees in the Crushed treatment were significantly more likely to exhibit gut melanization and hindgut expansion, providing support for the hypotheses that additional chemical and physical defenses were present. In particular, the chemical hypothesis is supported by our finding that larger bees in the Chemistry treatment had significantly more area of melanization than bees in other treatments.

Overall our study provides evidence that pollen defenses impact both larval and adult bees, through pre-ingestive and post-ingestive effects. Bees were deterred from feeding on cucurbit pollen both in nature and in our lab experiment, suggesting some cue indicates the suitability of the pollen diet for consumption. When feeding on the cucurbit diet, we found that microcolonies suffered severe fitness effects of both increased mortality and reduced reproduction. Deterrence mechanisms in this system could thus serve as honest signals of defense allowing bees to avoid physiological damage caused by ingesting defended pollen. Particularly if pre-ingestive defenses are less costly for plants when compared to post-ingestive defenses, this may be an efficient mechanism to reduce pollen loss while minimizing costly defenses. We also found that secondary plant chemicals in cucurbit pollen and DMSO may act as chemical defenses by reducing the digestibility of nutrients in pollen, but more study is needed to verify this finding. Ultimately, it would appear that cucurbit pollen is not an optimal diet for the generalist bumble bee *B. impatiens*. Our results indicate that cucurbit pollen bears physical, nutritional, and possibly chemical, defenses that are capable of imposing severe physiological costs on both adult and larval *B. impatiens*, in contrast with the specialist squash bee (*E. pruinosa*) which utilizes cucurbit pollen as its sole pollen source without ill effect. Our results are consistent with the hypothesis that different combinations of these pollen traits could allow plants to selectively attract and deter particular suites of pollinators that have physiological adaptations to different defenses. Future research should be directed at how widespread pollen defenses are, and how they may shape the evolution of pollinator floral preferences. Understanding how generalist bees respond to pollen defenses can provide new insights into digestive adaptations to the pollen diet as well as elucidate the context for trade-offs between diet generalization and specialization.

## Methods

### Assessing pollen collection

In the Finger Lakes Region of New York in 2012 and 2013, we supplemented cucurbit fields (0.5–10 ha, various pumpkin cultivars of *C. pepo*) with commercially produced *B. impatiens* colonies (Koppert Biological Systems, Inc.) or with locally rented *A. mellifera* hives. Sampling was conducted from 16 July-27 August in 2012 in ten fields (*n* = 4 honey bee supplemented, *n* = 6 bumble bee supplemented), and from 15 July-21 August in 2013 in ten fields (*n* = 5 honey bee, *n* = 5 bumble bee). Pollen from the corbiculae was collected from ten bees returning to the colonies during each of three rounds of sampling (total of *n* = 30 individuals per field). Bees were sampled between 0600–1200 h, during the time when *C. pepo* flowers were open. Bees were captured by aerial net, and pollen from their corbicula was removed, placed into a centrifuge tube with 150 µL of 95% ethanol and placed on ice for transport to the lab. Each sample was first mixed by pipetting; then a drop of this mixture was applied to a microscope slide, covered with Calberla’s fluid (recipe in Supplemental Materials), melted on a hotplate, and sealed with clear nail polish. For each slide a random subsample of 100 pollen grains was counted under a compound light microscope and identified to the lowest possible taxonomic unit (family or genus, with the exception of *Zea mays*) using a reference library of pollen created from local concurrently blooming plant species. Pollen was counted from a total of 315 bumble bees and 341 honey bees.

In the Finger Lakes Region of New York in August 2014 female *E. pruinosa* (*n* = 37) were collected while foraging on cucurbit plants from seven sites, (six of which were adjacent to *E. pruinosa* nesting aggregations). Bees were washed in ethyl acetate to remove pollen, which was then slide-mounted in Calberla’s fluid (recipe in Supplemental Materials). We performed six randomly selected field of view transects across the slide at 20X magnification, counting all grains except those that were broken or incomplete. Pollen was then identified to the lowest taxonomic rank feasible (family or genus, with the exception of *Zea mays*).

### Microcolony experimental protocol

From March-May 2017, we used a microcolony protocol to test whether *B. impatiens* was negatively impacted by consuming cucurbit pollen. This technique has been successfully used in previous studies to assess the effects of diet on bee colony performance^47,48,69–71^. Our experiment was replicated using three source colonies of *Bombus impatiens* (Biobest Canada Ltd. Leamington, ON, Canada). Each source colony supplied three microcolony replicates for each of five treatments. Microcolonies consisted of five workers taken from a source colony at least 24 hours after adult eclosion and were kept in a growth chamber at 27 °C and 60–80% humidity. We attempted to standardize bee size as much as possible within a microcolony, but limitations due to the number and size of bees eclosing from source colonies on any given day meant that there was variation in the size of individual bees and the average weight of microcolonies. We initially fed bees a mixture of a standard diet of honey bee collected pollen (CC Pollen Company High Desert Fresh Raw Bee Pollen Granules) that was determined to be pesticide-free^72^ and 30% sucrose solution, while they acclimated to the microcolony for two-six days and established a dominance hierarchy^47,69^. After this period, one bee typically develops into a pseudoqueen capable of laying eggs.

Sucrose solution (30% in 1/2 oz cups) and pollen (mixed with 30% sucrose solution according to treatment, detailed below, in 0.3 g portions) were provided *ad libitum*. Treatments were produced in bulk before the start of the experiment and then stored at −20 °C until use. Cucurbit pollen was collected from plants grown in a greenhouse at Cornell University (in July-August 2016 and January-February 2017), in order to acquire pollen from non-herbivore damaged, pesticide-free plants. Previous work has shown that cultivars of *Cucurbita pepo* show variability in pollen chemistry (Brochu unpublished data), so the pollen of several cultivars was mixed for the cucurbit pollen diet (see Supplementary Table S1 for proportions and varieties used). We used five treatments (Table 1) to differentiate between the effects of chemical and physical traits as well as nutrition: (1) Diet Control, (2) Solvent Control, (3) Added Chemistry, (4) Crushed Cucurbit, and (5) Natural Cucurbit (See Supplementary Fig. S3 for photos).

**Table 1:** Summary of diet treatments provided to *B. impatiens*. Each microcolony consisted of 5 worker bees. Treatments were replicated three times for each source colony for a total of 9 replicates across colonies.

Treatment Name	Treatment Contents	Possible Chemical Defenses	Possible Poor Nutrition	Possible Physical Defenses
Diet Control	Standard pollen diet + 30% sucrose			
Solvent Control	Standard pollen diet + 5%DMSO in 30% sucrose			
Added Chemistry	Standard pollen diet + extracted cucurbit chemistry dissolved in 5% DMSO in 30% sucrose	X		
Crushed Cucurbit	Homogenized cucurbit pollen + 30% sucrose	X	X	
Natural Cucurbit	Unmanipulated cucurbit pollen + 30% sucrose	X	X	X

The natural treatment consisted of pure cucurbit pollen, which retained physical traits that could act as physical defenses, chemicals that could act as chemical defenses, and may not have been nutritionally sufficient for *B. impatiens* development. Natural treatment pollen consisted of unmanipulated cucurbit pollen diet mixed with 30% sucrose solution. The crushed treatment consisted of crushed cucurbit pollen to eliminate physical traits that could act as defenses, but still retained chemicals that could act as defenses, and additionally, may not have been nutritionally sufficient for *B. impatiens* development. Cucurbit pollen was bead homogenized into water using a FastPrep-24 Classic Instrument (M.P. Biomedicals, USA) twice for 30 s at 4.0 m/s, with the water evaporated using an N-EVAP 112 Nitrogen Evaporator (Organomation, MA, USA), and then mixed with 30% sucrose solution to obtain the Crushed cucurbit treatment. The chemistry treatment contained chemicals extracted from cucurbit pollen into a solvent on the standard pollen diet (described above), which eliminated physical traits with the potential to act as defenses and should have been nutritionally sufficient, but may have contained chemicals acting as defenses. The cucurbit chemical extract was obtained by bead homogenization of the cucurbit pollen diet into methanol using a FastPrep-24 Classic Instrument (M.P. Biomedicals, USA) twice for 45 s at 6.5 m/s. The methanol was then evaporated using a CentriVap Benchtop Vacuum Centrifuge (Labconco Corporation, MO, USA) and the chemical extract was resuspended in a 5% DMSO solution in 30% sucrose solution. This solution was then mixed with the same amount of standard pollen diet as the amount of cucurbit pollen diet used in the extraction. The solvent control contains the solvent (DMSO) on the standard pollen diet to control for potential negative effects of the solvent when assessing the chemistry treatment, and the diet control treatment is a multi-floral diet with pollen traits that are unlikely to act as defenses, as it is sufficient for *B. impatiens* growth and reproduction. Solvent and Control treatment pollen consisted of the standard pollen diet mixed with 5% DMSO in 30% sucrose solution and just 30% sucrose solution, respectively. Treatment pollen was added two to six days after the microcolony was formed.

Nectar refills were monitored to assess how much sucrose the bees were consuming. Pollen was weighed daily to record bee consumption. Pollen for each treatment was also maintained outside microcolonies and weighed daily in order to control for weight loss due to evaporation. The following measures of fitness were recorded daily: (1) number of dead workers, (2) number of larval cells produced, and (3) number of ejected larvae. Microcolonies were terminated once the first adult offspring eclosed or at 50 days from inception, whichever came first. Following termination, all adult bees including newly eclosed offspring were weighed and then euthanized by freezing. These bees were later dissected to observe gut morphology changes as a result of the treatments. Melanization (hardened and darkened portions of the gut) was observed largely in the midgut, while hindgut expansion (swollen and ballooned) was possibly due to difficulty in passing the diet treatment. Melanization and hindgut expansion were assessed qualitatively (presence/absence) and quantitatively, comparing the size of the affected area using the image analysis software, Fiji v1.52i^73,74^.

Microcolonies were allowed to remain intact for 24 hours following termination to allow for the emergence of any additional adult offspring, and following this incubation period they were assessed for total reproductive output. All remaining larval offspring were counted and weighed, then euthanized by freezing.

### Statistical analyses

All analyses were conducted in R version 3.6.0 (https://www.r-project.org/) using the following packages: AICcmodavg, car, coxme, emmeans, ggplot2, ggpubr, influence.ME lme4, MASS, MuMIN, plyr, rcompanion, reshape2, RVAideMemoire, spaMM, survival, survminer and the HighStat Library^75–92^.

We used the ‘lmer’ function^76^ to fit linear mixed effects models for pollen consumption per bee per day, average sucrose consumption per bee per day, pollen efficiency (number of eclosed offspring/total pollen consumed per microcolony), area of hindgut per gram of bee body mass, and area of melanization. Proportion of cucurbit pollen collection, proportion of adult mortality, probability of raising offspring to adulthood, number of ejected larvae per bee per day, proportion of bees with hindgut expansion, and proportion of bees with melanization were assessed with the glmer and glmer.nb functions^76^ to fit generalized linear mixed effects models with binomial, poisson (for count data), or negative-binomial (in the case of over-dispersed data, indicated in the text) distributions. All full models included Treatment, Average Weight (microcolony) or Weight (individual bee), and their interaction as fixed effects and replicate within source colony as random effects, unless otherwise indicated below. We confirmed the absence of multicollinearity in our predictors using the function ‘corvif’^85^. For each analysis we selected the best model by removing non-significant effects and comparing models with the ‘anova’ function. If no fixed effect was significant, we compared all possible models using the ‘dredge’ function^75^. Best models are described in Table S2. Best models were fit using restricted maximum-likelihood (REML) and Kenward-Roger approximations for degrees of freedom are reported and used to evaluate significance^93^.

The model for proportion of cucurbit pollen collected included only species as a fixed effect and site within year as random effects. Pollen consumption was standardized to consumption per bee for each day of the experiment and compared over time using a repeated measures mixed effects regression with a fully crossed fixed effects design. We used the same method to assess the mass lost to evaporation and used these results to correct our measures of pollen consumption (see Supplemental, Table S3 and Fig. S4, for more details). Day of experiment was the repeated measure and was added to these models as a continuous fixed effect. Pollen consumption was natural log plus 0.0012 (the minimum value) transformed to improve normality of model residuals. Sucrose consumption was not monitored daily, and was thus assessed as average sucrose consumption per bee per day. We used the ‘coxme’ function^80^ to fit a Cox Proportional Hazards mixed effects model, to assess the probability of individual bee mortality over 27 days. This time period was before the first microcolony was terminated due to the production of an adult offspring and avoided biasing our analysis with differential termination dates. Treatment, Average Weight, and their interaction were included as fixed effects and replicate within source colony as random effects. The model for probability of raising offspring to adulthood did not converge due to issues of separation in the data, thus the effect of Treatment was assessed with Fisher’s Exact test.

We used the ‘emmeans’ function^78,94^ to conduct all post-hoc tests. For models with interaction effects between Treatment and individual bee Weight, we conducted post-hoc tukey tests to compare treatment effects across seven weight classes (minimum, 5^th^ percentile, 25^th^ percentile, median, 75^th^ percentile, 95^th^ percentile, maximum). For analyses at the microcolony level, each treatment consisted of nine replicates for a total of 45 microcolonies. For the pollen consumption analysis, pollen consumption was assessed for each microcolony, daily, until it was terminated, resulting in 1694 observations (due to differential termination dates). For analyses at the individual bee level (mortality over time and proportion data), each treatment included 45 bees for a total of 225 bees. For the dissection analyses, two bees had digestive tracts that could not be dissected, thus the analysis of hindgut area was restricted to 223 bees. Only 60 bees exhibited melanization on their digestive tract, thus the analysis of area of melanization was restricted to this subset of data.

## Supplementary information

Supplementary information

## Data Availability

Data are available from the Dryad Digital Repository: 10.5061/dryad.gb5mkkwks.
