# Supplementary material for: Pollen defenses negatively impact foraging and fitness in a generalist bee (Bombus impatiens: Apidae)
Source: Sci Rep. 2020 Feb 20;10:3112. doi: 10.1038/s41598-020-58274-2 (PMC7033150; doi:10.1038/s41598-020-58274-2)

**Supplementary Materials**

Figure S1. Effect of average weight on sucrose consumption.


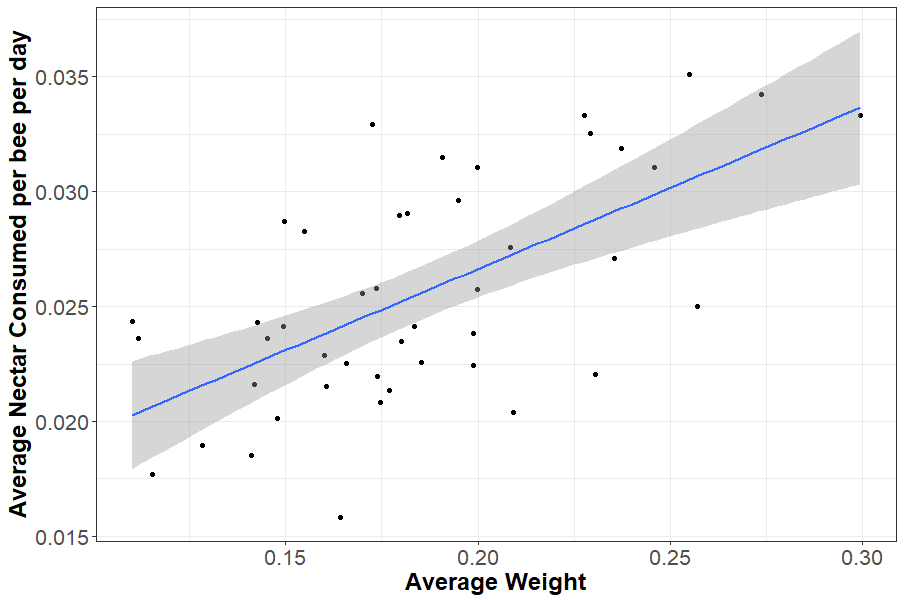


Figure S2. Effect of treatment on pollen efficiency


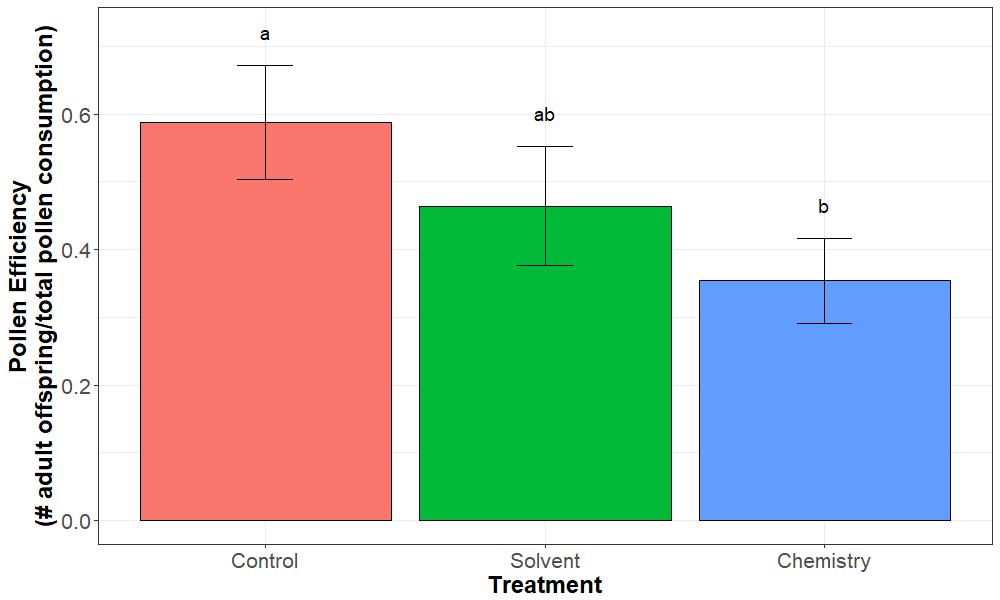


*Calberla’s Fluid recipe*

Calberla’s Fluid consisted of 5 ml glycerol, 10 ml 95% ethanol, 15 ml distilled water, and 3 to 4 drops saturated aqueous basic fuchsin.

Table S1. Varieties of *Cucurbita pepo* used in the Cucurbit pollen diet mix.

| **Variety** | **Fruit** | **Percentage** |
| --- | --- | --- |
| Black Beauty | Zucchini | 11.68 |
| Baby Pam | Pie Pumpkin | 7.05 |
| Cougar | Summer Squash | 3.08 |
| Flying Saucer | Patty pan Squash | 6.77 |
| Golden Arrow | Summer Squash | 6.26 |
| Howden | Carving Pumpkin | 15.89 |
| New England Pie | Pie Pumpkin | 16.33 |
| Success PM | Summer Squash | 13.61 |
| Tom Fox | Carving Pumpkin | 11.60 |
| Zephyr | Summer Squash | 7.73 |

Figure S3. Photographs of (a) Natural Cucurbit pollen and (b) Crushed Cucurbit pollen treatment at 100X magnification.

**(b)**

**(a)**

| 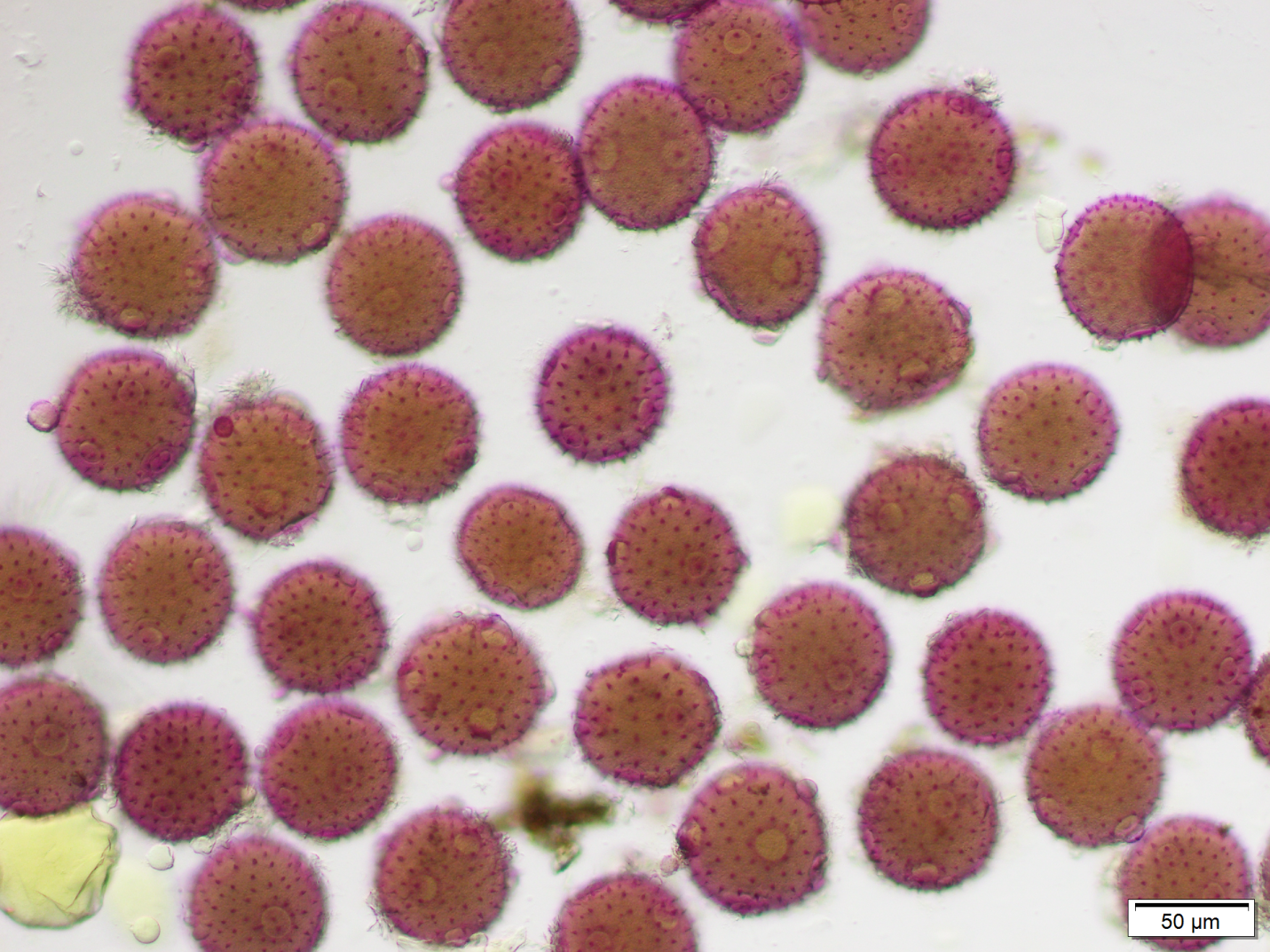 | 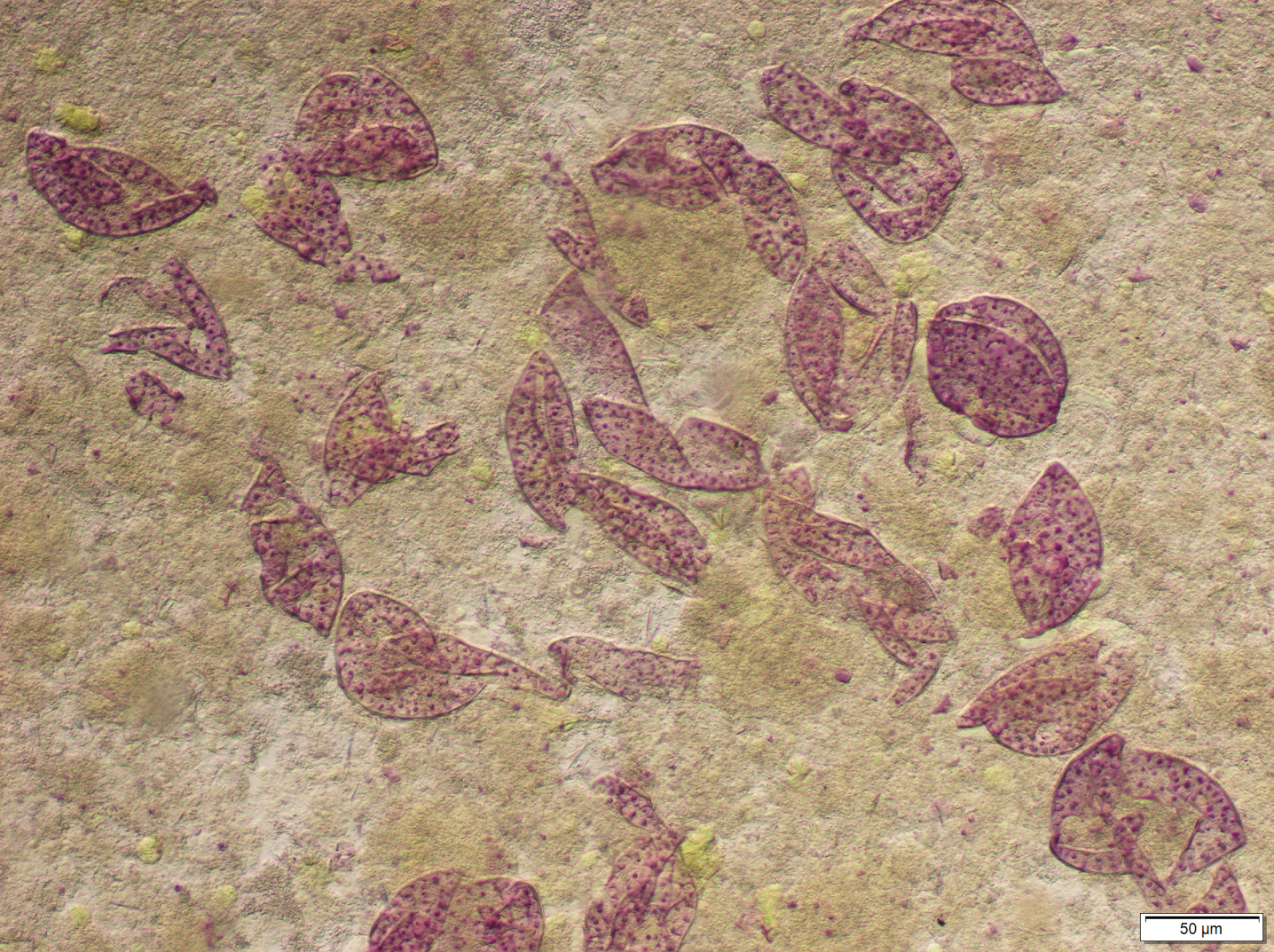 |
| --- | --- |

*Linear Model Details*

The models for average sucrose consumed per bee per day, days to first eclosed offspring, number of larvae ejected per bee per day, proportion of hindgut expansion, and the amount of melanization had singular fits due to difficulty estimating some of the random effects parameters. Models run without these random effects produced the same results for all models affected by singular fits. The model for the probability of producing adult offspring would not converge due to semi-perfect separation in the data, therefore the data was analyzed using Fisher’s Exact test.

The models for the number of days to the first eclosed offspring and the average eclosed worker weight both had no significant fixed effects and the models with the lowest AICc score were the intercept-only models. The model for the number of eclosed offspring was underdispersed and subsequently analyzed with a ComPoisson model with the number of days of worker bee activity as an additional fixed effect, using the function ‘HLfit’ in the Rpackage ‘spaMM’ ^1^. No significant fixed effects were found.

*References*

1. Rousset, F. & Ferdy, J.-B. Testing environmental and genetic effects in the presence of spatial autocorrelation. *Ecography* **37**, 781–790 (2014).

Table S2. Detailed results for statistical tests performed. Fixed effects listed represent the optimal model as indicated by AICc scores. Bold indicates significance at p < 0.05, italics indicates significance at p < 0.1.

| **Response Variable** | **Fixed Effects** | **Statistic** |
| --- | --- | --- |
| 1) Proportion Cucurbit Pollen Collected | **Species** | *Χ^2^*_(2)_ = 112.11, **p < 0.001** |
| 2) Untransformed Pollen Consumption per bee | Treatment  **Day**  Average Weight  **Treatment : Day**  Treatment : Average Weight  **Day : Average Weight**  **Treatment : Day : Average Weight** | F_(4,1662)_ = 0.686, p = 0.602  F_(1,1671)_ = 69.559, **p < 0.001**  F_(1,1262)_ = 0.019, p = 0.891  F_(4,1670)_ = 15.490, **p < 0.001**  F_(4,1658)_ = 0.344, p = 0.848  F_(1,1671)_ = 47.294, **p < 0.001**  F_(4,1669)_ = 18.226, **p < 0.001** |
| 3) The natural log of Pollen Consumption per bee + 0.0012 | Treatment  **Day**  *Average Weight*  **Treatment : Day**  Treatment : Average Weight  **Day : Average Weight**  **Treatment : Day : Average Weight** | F_(4,1666)_ = 0.6817, p = 0.605  F_(1,1671)_ = 86.304, **p p < 0.001**  F_(1,1362)_ = 2.764, *p = 0.097*  F_(4,1669)_ = 20.741, **p < 0.001**  F_(4,1664)_ = 0.619, p = 0.649  F_(1,1670)_ = 81.280, **p < 0.001**  F_(4,1669)_ = 18.658, **p < 0.001** |
| 4) Average Sucrose Consumption per bee per day | **Average Weight** | F_(1,14)_ = 16.001, **p = 0.001** |
| 5) Proportion Adult Mortality | **Treatment** | *Χ^2^*_(4)_ = 46.628, **p < 0.001** |
| 6) Number of Ejected Larva per bee per day | **Treatment** | *Χ^2^*_(4)_ = 16.804, **p = 0.002** |
| 7) Pollen Efficiency | **Treatment** | F_(2,14)_ = 5.310, **p = 0.019** |
| 8) Probability of Hindgut Expansion | **Treatment** | *Χ^2^*_(4)_ = 19.087, **p < 0.001** |
| 9) Area of Hindgut per gram of bee body mass | **Treatment** | F_(4,210)_ = 5.432, **p < 0.001** |
| 10) Probability of Melanization | **Treatment** | *Χ^2^*_(4)_ = 31.148, **p < 0.001** |
| 11) Area of Melanization | Treatment  Weight  **Treatment:Weight** | F_(4,48)_ = 0.429, p = 0.787  F_(1,50)_ = 0.111, p = 0.740  F_(4,47)_ = 3.125, **p = 0.023** |

*Mass loss due to evaporation*

We found that the Crushed and Natural cucurbit pollen lost more mass due to evaporation than the other treatments, but only on day one (Table S1, Figure S1). Mass loss due to evaporation was not significant from days two through seven. We thus corrected daily pollen consumption for microcolonies fed the Crushed and Natural cucurbit treatments by the mean value lost to evaporation only on the first day of replacement.

Table S3. Summary of analysis for pollen mass lost to evaporation.

| **Response Variable** | **Fixed Effects** | **Statistic** |
| --- | --- | --- |
| Pollen Mass Lost to Evaporation | Time Interval  **Treatment**  **Treatment : Time Interval** | F_(6,1)_ = 0.916, p = 0.649  F_(4,571)_ = 22.091, **p < 0.001**  F_(24,569)_ = 17.042, **p < 0.001** |

Figure S4. Mass loss due to evaporation over time by treatment.


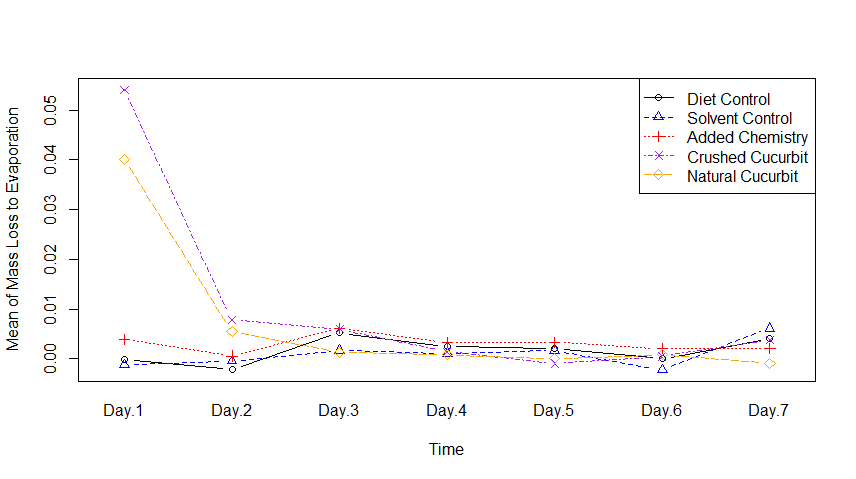

Supplement: Supplementary file 1 — Supplementary information [file 41598_2020_58274_MOESM1_ESM.docx]
